# Gamification and motivation: Impact on delay discounting performance

**DOI:** 10.1371/journal.pone.0299511

**Published:** 2024-04-16

**Authors:** Sophie Harvey, Greg Jensen, Kristen G. Anderson

**Affiliations:** 1 Digital Humanities, University of Alberta, Edmonton, Alberta, Canada; 2 Department of Psychology, Reed College, Portland, Oregon, United States of America; University of Zagreb Faculty of Electrical Engineering and Computing: Sveuciliste u Zagrebu Fakultet Elektrotehnike i Racunarstva, CROATIA

## Abstract

Delay discounting is a phenomenon strongly associated with impulsivity. However, in order for a measured discounting rate in an experiment to meaningfully generalize to choices made elsewhere in life, participants must provide thoughtful, engaged answers during the assessment. Classic discounting tasks may not optimize intrinsic motivation or enjoyment, and a participant who is disengaged from the task is likely to behave in a way that provides a biased estimate of their discounting function. We assessed degree of delay discounting in a task intended to vary level of participant motivation. This was accomplished by introducing varying levels of *gamification*, the application of game design principles to a non-game context. Experiment 1 compared three versions of the delay discounting task with differing degrees of gamification and compared performance and task enjoyment across those variations, while Experiment 2 used two conditions (one gamified, one not). Participants found more gamified versions of the task more enjoyable than the other conditions, without producing substantial between-group differences in most cases. Thus, more polished task gameplay can provide a more enjoyable experience for participants without undermining delay discounting effects commonly reported in the literature. We also found that in all experimental conditions, higher levels of interest in or enjoyment of the task tended to be associated with more rapid discounting. This may suggest that low task motivation may result in less impulsive choice and suggests that participants who find delay discounting experiments sufficiently boring may bias assessments of value across delays.

## Introduction

Delay discounting (DD) is a pattern of preference where participants demonstrate that the value of a reward decreases as a function of how far into the future it is delayed. Studies using DD paradigms have shown that both humans and animals tend to prefer smaller, more immediate rewards over larger, delayed rewards [1, 2]. The *delay discounting rate* governs how rapidly a reward’s value shrinks as its delay into the future grows. Participants with a high rate will generally prefer small immediate rewards over much larger rewards delivered with even a moderate delay. On the other hand, low discounting rates correspond with willingness to forego small immediate rewards in favor for larger delayed rewards, even if delays are considerable. Consequently, DD has been used as a method of not only evaluating impulsive behavior but also as a model of decision making.

Tasks that measure DD have been used to evaluate impulsivity and decision making with respect to both hypothetical and real money [3], commodity values [4], and even abstract experiences [5]. An important focus has been drugs of abuse. For example, Bickel, Odum, and Madden [6] used DD to measure the comparative impulsivity of smokers, ex-smokers, and non-smokers. The authors used hypothetical monetary rewards for all groups in addition to cigarette rewards for the smoker group. They found that not only did smokers discount money more than both ex-smokers and non-smokers, they also discounted cigarettes more than the monetary rewards. Consequently, Bickel and colleagues concluded that those with drug dependence are more likely to show a greater degree of discounting. Recent work suggests core deficits among individuals with opioid dependence, indicated by more frequent selection of nonoptimal choices and relative impairment in discriminating optimal from nonoptimal responses when compared with controls on a novel DD paradigm [7]. Discounting differences among individuals with alcohol and other drug use disorders have been broadly replicated with other addictive substances (for review, see [8]) and problematic use of video games [9].

Delay durations and reward size are not the only task variables that impact discounting. According to a recent meta-analysis, variations in experimental procedure substantially impact estimated discounting rates [10]. Across ninety-two papers, the authors identified nine different manipulation types. Ten studies focused on *framing effects*, which occur when descriptions of either time or the outcome described for a task are framed in terms of some hypothetical scenario. Even superficial changes, such as describing delays in terms of days, weeks, or months, resulted in changes in measures of discounting [11]. While this sensitivity to procedure does not necessarily undermine the diagnostic value of laboratory tasks, concerns are nevertheless raised that discounting measured in the lab may provide a biased estimate of real-world decision tradeoffs.

A criticism of laboratory measures, like DD, is that their poor ecological validity negatively impacts motivation [12]. Scherbaum et al. [7] provided anecdotal evidence that classical DD paradigms were "boring and not very engaging" as reported by participants, resulting in low task motivation (p. 146). Self-determined, autonomous motivation is believed to be the optimal form of motivation and involves voluntary engagement in activities that provide reinforcement by meeting needs for enjoyment or internal goal-striving. This intrinsic motivation results in improved performance in many psychological tasks [13]. As such, classical DD paradigms which focus solely on external factors (such as feeling a social obligation to complete the task) may not activate this key motivational domain.

DD tasks have in some cases been embedded into more elaborate video game experiences [7, 14]. This gamification of the DD task has the potential to increase motivation, in turn giving a better characterization of the kinds of discounting that participants will make in real-life scenarios with meaningful stakes, as well as allowing longer protocols in which more data can be collected. Importantly, gamification represents a more involved design process that introduces both game-like interactions and a game-like aesthetic. Gamification is defined as “the use of game design elements in non-game contexts” (p.10) [15]. Certain game elements can be isolated and applied to non-gaming contexts to increase task motivation [15], particularly to the benefit of activities that otherwise seem boring or onerous. In the healthcare context, Garett and Young [16] found that the most popular game elements were points, social interactions, leaderboards, and progress status. These common gamification elements have proved fruitful in educational contexts [17] and employment contexts [18]. Gamification can introduce additional features to a task that can act as discriminative stimuli, in that they evoke behavior more reliably than if they were absent [19]. For example, because gamification encourages the player to treat the target activity as a game, the task may elicit higher motivation, greater curiosity, and even a more enjoyable experience.

However, changes to the task need not be substantive to impact user interest; Lieberoth’s [20] *framification hypothesis* states that simply framing a task as a game, even without having game mechanics or complex representative graphics, can still impact engagement. If tasks can be gamified to increase motivation without affecting their reliability and validity, rates of task completion may improve and provide better indices of performance in the laboratory or clinic. Several studies have attempted to explore the impact of gamification on cognitive tasks. A 2016 review of gamification of cognitive tasks found that those studies utilizing a measure of intrinsic motivation in their experiment reported that gamification improved motivation on tasks when compared to non-gamified versions [21].

Gamification has also negatively impacted participant attitudes in some cases. Birk et al. [22] isolated several psychological tasks and gamified them by adding a premise and graphics. They reported that gamification decreased precision and accuracy in a go/no go task, as compared to an unmodified version that elicited higher levels of enjoyment, autonomy, and immersion. Friehs et al. [23] gamified aspects of the stop-signal task, another index of response inhibition, in two experiments. They found no differences in task performance between the two conditions. When conditions were compared using a within-subjects design, participants rated the gamified version higher on the Interest/Enjoyment subscale of the Intrinsic Motivation Inventory (IMI) [24]; however, no such difference materialized in a between-subjects design. This suggests that gamification only increased the enjoyment of the stop-signal task when participants could compare the two conditions. As such, whether gamification improves motivation should not be assumed; it is an empirical question that should be assessed for a given paradigm before that approach is widely adopted.

In this investigation, we focused on the DD task as a target for gamification given its common usage in psychopathology research, validation against other indices of impulsivity, and potential amenity to gamification given the task parameters. Two experiments manipulated key game elements of framing and integration of game elements, in order to determine whether gamification would result in enhanced intrinsic motivation for participants. Additionally, we were curious whether or not gamification of DD would result in a systematic shift in discounting rates, which could complicate the comparison of gamified and non-gamified DD estimates. The second experiment broadened our sample and provided the opportunity to better examine which participants might be more susceptible to impacts on motivation.

## Experiment 1

Three versions of the DD task with differing levels of gamification (Control, Framing, and Gamified) were developed and successfully implemented to evaluate how the original DD task compares to versions with different gamified elements. The Framing condition was based upon Lieberoth’s [20] framification hypothesis that simply framing a task as a game, even without having game mechanics, can still have an impact on engagement. Based on previous research [21, 23], we anticipated that both the Gamified and Framing conditions would demonstrate higher levels of intrinsic motivation, specifically enjoyment, than the control condition with the core impulsivity estimates from the DD task remaining the same.

### Materials & methods

#### Participants

A total of 100 participants, aged 24 to 92, completed the experiment (two participants chose not to report their age). Thirty-two who identified as women and 68 who identified as men were recruited from Amazon’s MTurk. The racial makeup was predominantly White (*n* = 67), but also included Asian or Pacific Islanders (*n* = 18), Black/African Americans (*n* = 8), Latinx/Hispanics, (*n* = 4), Native Americans (*n* = 2), and one participant who identified as Other. The overall mean score on the Game Genre Questionnaire was 1.95 (meaning that most participants had some experience with video games), with the most frequently played genres being shooters (*M* = 2.52, *SD* = 1.06), platformers (*M* = 2.47, *SD* = 0.94), and action role playing games (*M* = 2.43, *SD* = 1.09).

Participants were recruited through an advertisement posted on MTurk from 10/12/17 to 10/18/17. When recruited, participants were informed that they would be completing a survey about their opinions and preferences, and that no published data would include identifying information. Participants were also provided with the contact information for the institutional review board (IRB). Once this information was provided, participants gave written consent to continue; those who did not provide this written consent were not recruited into the study. In exchange for their responses, participants received $1 in compensation.

After obtaining consent, participants completed demographic items, the Game Genre Questionnaire, and the UPPS-P. Participants were then randomly assigned to complete one of three tasks: Control (*n* = 35), Framing (*n* = 26), or Gamified (*n* = 39). Subsequent to finishing the DD procedure, participants completed the IMI [24] and were compensated $1.00 for their time. Anonymized data from Experiment 1 is available here {https://psyarxiv.com/6xcbe/}.

All procedures, stimuli, and survey materials were approved by the Reed College IRB to assure ethical treatment of participants (protocol no. 2017-F1).

#### Measures

Participants completed a demographics survey that included questions about age, sex (i.e., *Male*, *Female*, *Other*, *Prefer not to answer*), and ethnicity (*Asian or Pacific Islander*, *Black/African American*, *Caucasian/White*, *Latinx/Hispanic*, *Other*, *Prefer not to answer*).

The Game Genre Questionnaire (GGQ; unpublished undergraduate thesis, OCLC number: 988095403) is a 32-item self-report scale evaluating video game engagement across genres. Participants were provided with a list of game genres (and example games of each genre) and asked to respond how often they play each type of game on a scale of *Never*, *Seldom*, *Occasionally*, and *Frequently* (coded as 1, 2, 3, and 4, respectively). This measure was successfully used in a small sample of undergraduates in previous gaming research and was included here to assess participants’ familiarity and experience with interactive games.

The UPPS-P Impulsive Behavior Scale [25] is a 59-item self-report scale developed to assess impulsivity along multiple dimensions: sensation seeking (α = .90), the tendency to seek out and enjoy novel experiences; lack of premeditation (α = .88), the inclination to act without considering the risks or consequences; lack of perseverance (α = .88), the inability to remain focused on a given task; negative urgency (α = .91), the tendency to engage in risky behaviors when under negative emotions; and positive urgency (α = .95), the tendency to engage in risk behaviors when under positive emotions. The UPPS-P has been validated in adult populations across genders [26] and showed excellent reliability in this sample.

The Task Evaluation Questionnaire is a 22-item subscale of the Intrinsic Motivation Inventory (IMI) [24], a self-report questionnaire designed to assess a participant’s experience in a target activity and asked to rate how true a statement is for them on a scale from 1 (*not true at all*) to 7 (*very true*). Four pertinent subscales assess aspects of the task including interest/enjoyment (α = .95), perceived competence (α = .82), perceived choice (α = .85), and pressure/tension (α = .79). The IMI has shown strong reliability and validity in adult samples [24] and demonstrated good to excellent internal consistency in this sample as seen above.

#### Procedure

**Fig 1:**
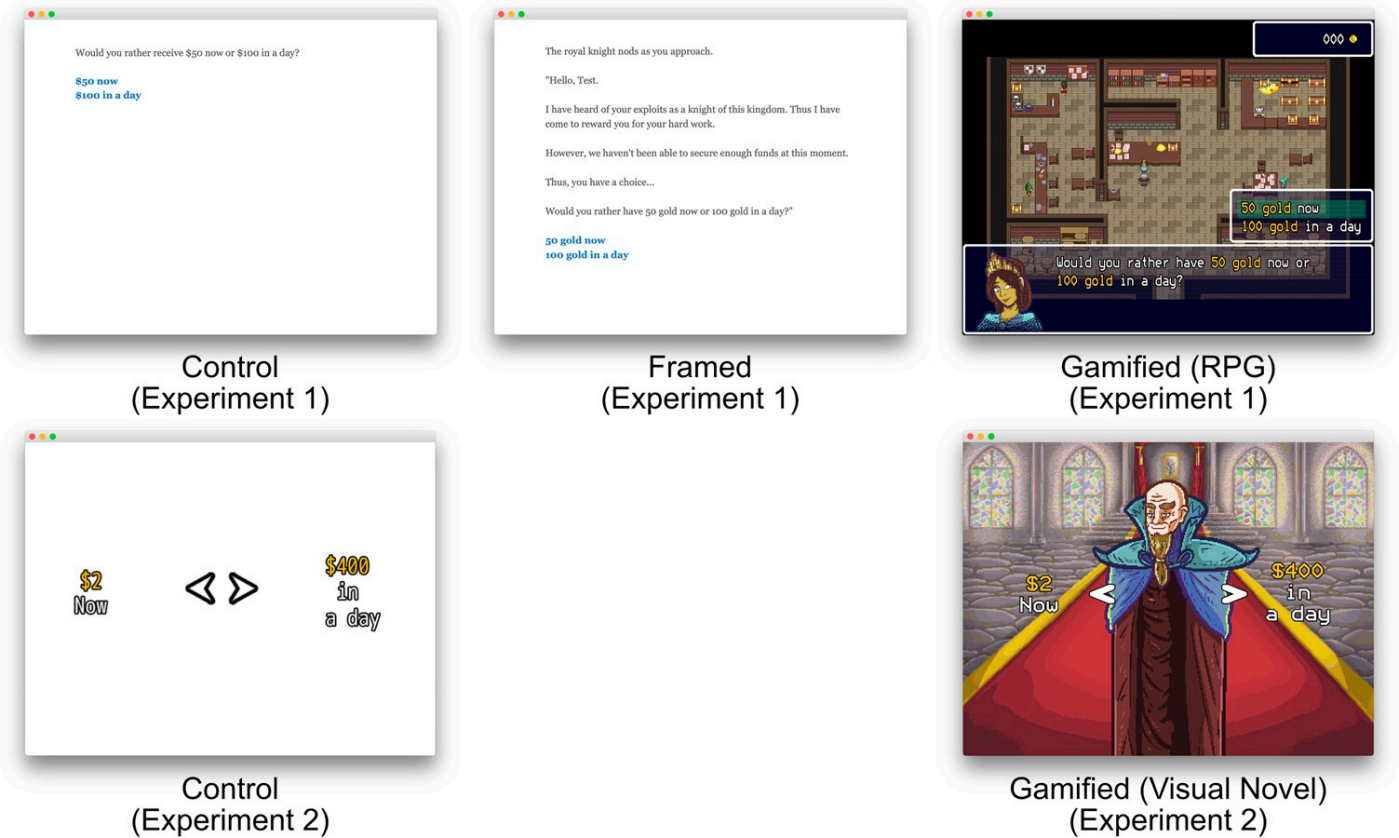
(Top Row). Representative screens from the control condition (left), the framing condition (center), and the gamified condition (left) in Experiment 1. Reproduced with permission (https://doi.org/10.6084/m9.figshare.24953352.v1). **(Bottom Row).** Representative screens from the control condition (left) and the gamified condition (right) in Experiment 2). Reproduced with permission (https://doi.org/10.6084/m9.figshare.24953379.v1).

The task required participants to choose between two hypothetical amounts of currency, either available now or after one of four time periods: a day, a week, a month, and three months (i.e., “Would you rather receive $50 now or $100 in a day?”). The Control condition (Fig 1, Top Left) replicated a typical DD study where participants are asked to choose between a hypothetical smaller, immediate sum of money or a delayed, larger sum of money. The Framing condition (Fig 1, Top Center) utilized the same core narrative as the Control condition but framed with an added fantasy storyline where the player is given the role of a knight who must obtain money (gold) for their family by interacting with another character. Additional dialogue options were added such as conversing with another character and examining a room. Participants entered their name, later referenced throughout the task. In the Gamified condition (Fig 1, Top Right), the participants entered their name in addition to choosing between two avatars. The narrative and decisions were the same as the Framing condition, but with the added ability to use the avatar to move and interact with objects and a non-player character who presented the DD task. A small window containing the participant’s current amount of gold was added to the top right of the screen. The core DD procedure remained the same across conditions. The participants were first asked if they would rather have $50 (or 50 gold in the Framing and Gamified conditions) now or $100 in a day. If the participant selected $50, this portion of the task ended, and the participant moved onto the next section. If the participant selected $100, they were then asked if they would rather have $75 now or $100 in a day. This process continued with the immediate option increasing to $88, $94, and finally $97. The procedure was again repeated with a delay of one week, one month, and three months.

Both the Control condition and the Framing condition were programed in Twine [27] (version 2.1.2), an open-source software for constructing text-based interactive stories. The Gamified condition task was constructed in RPG Maker MV (Kadokawa Corporation LLC, 2015), a program for creating two-dimensional role-playing games.

In this study, we used the quasihypberbolic model [28]:

$$E\left(\frac{V}{A}\right)=\beta \delta^{D}\;\text{when}\;D>0,E\left(\frac{V}{A}\right)=1.0\;\text{when}\;D=0$$ (Eq 1)

Here, *V* is the size of the smaller, immediate reward, whereas *A* is the size of the larger, delayed reward whose delay *D* is measured in days. *β* and *δ* are both free parameters, with *β* acting similarly to an intercept while δ governs the speed with which the discounting curve shrinks. These curves describe a participant’s “indifference point” at any given delay, a hypothetical discounted value for the immediate option that is subjectively equal to the full-value option available at that delay [29]. Since the indifference point at any given delay cannot be observed directly, we inferred its value using a statistical model, estimated using a Bayesian framework that can accommodate uncertainty about its value [30]. Models were fit using the Stan modeling language and are available in S1 and S2 Data [31].

In order to compare overall discounting performance, we adopted parametric AUC [32] as our primary outcome measure. A robust theory of DD requires using parametric models, but differing models cannot be compared in terms of their parameters. Such comparisons become possible if a parametric “model AUC” is calculated, consisting of the area under the formal model’s discounting curve out to the maximum delay assessed. This does not require calculus; instead, one can calculate the value of the discounting function for small, uniformly spaced intervals and then sum over the resulting trapezoids. With finer slices, the sum converges on the true integral, to whatever precision is required.

## Results

A bootstrapping analysis of the GGQ and the UPPS-P pre-task surveys, corrected for multiple comparisons using the Hommel procedure [33], did not yield any significant differences between conditions, with one exception: participants in the Gamified condition scored significantly higher on the sensation seeking items of the UPPS-P than did participants in the Framing condition. On this basis, we concluded that participants were fairly well distributed across conditions. This analysis and the corresponding plot of the data are included in the electronic supplement.

**Fig 2:**
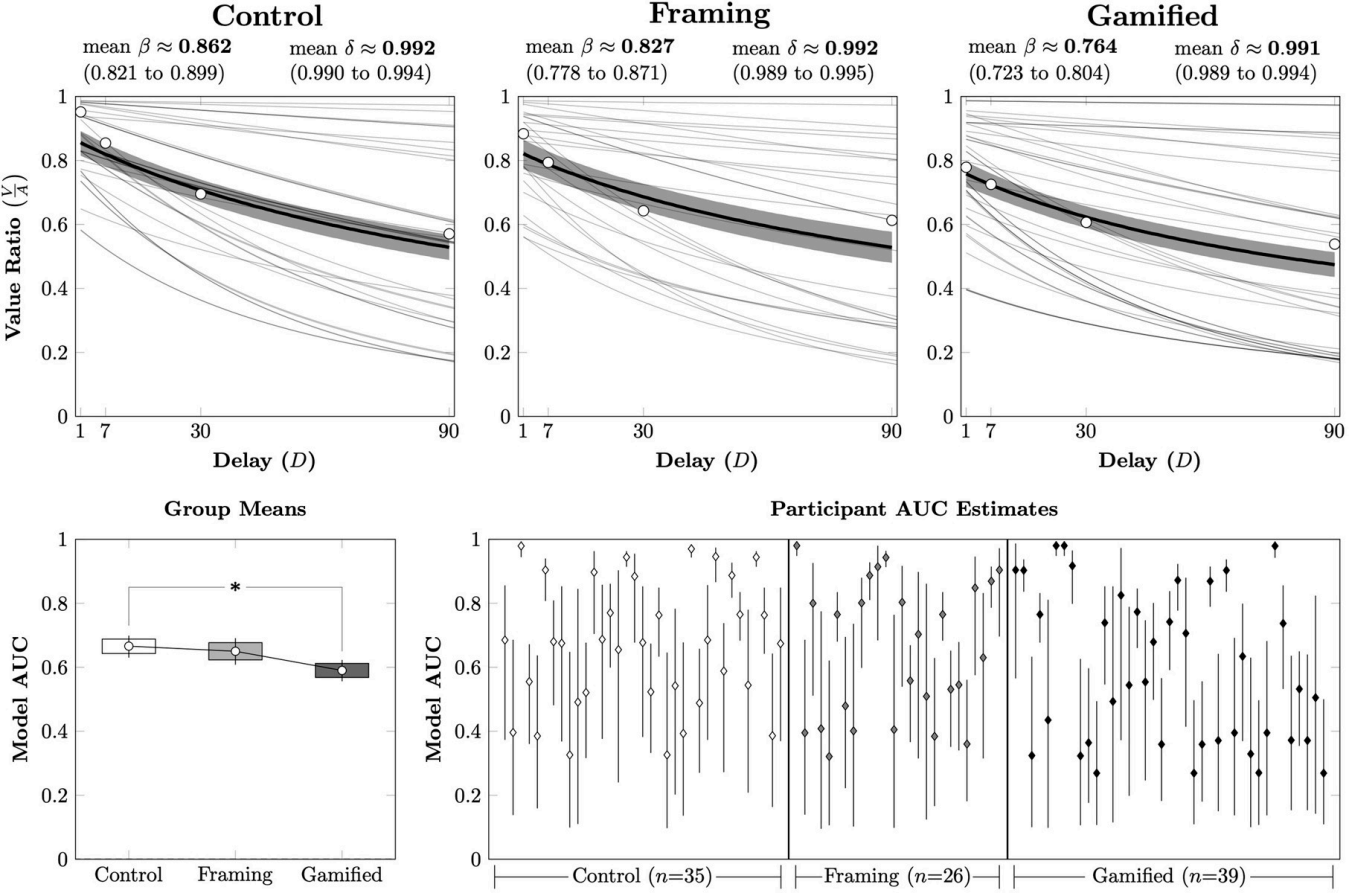
**Discounting behavior across participants in Experiment 1.** Error bars represent the 95% credible interval for the mean, while boxes represent the 80% credible interval. (*) represents a posterior distribution comparison that excludes zero from the 95% credible interval. **(Top Row)**. Posterior means for each participant’s estimated behavior, based on the quasihyperbolic discounting model in each of the three conditions. Thin gray lines represent participant estimates, whereas the thick black line represents the mean across participants in that condition, with shading depicting the 95% credible interval of that mean. Also plotted are the empirical mean across participants for the estimated indifference point at each delay. **(Bottom Left).** Mean parametric AUC across participants for each condition. **(Bottom Right)**. Estimated parametric model AUC for each participant, based on the posterior parameter estimates of the quasihyperbolic distribution.

Fig 2 (Top Row) plots the mean posterior quasihyperbolic discounting function for each participant in each of the three conditions as a function of the *V*/*A* ratio. Additionally, the overall mean discounting function across participants is plotted using heavy black lines, as are the mean estimated indifference points across participants. Participants displayed very similar discounting rates δ across conditions, but differed in their intercept parameters *β*, with the highest intercept (and thus least discounting) in the Control condition and the lowest intercept (and thus most discounting) in the Gamified condition.

Discounting was then characterized using a model AUC measure (using Equation 2 to numerically integrate the quasihyperbolic model), as depicted in Fig 2 (Bottom Left). Participants discounted significantly more on average in the Gamified condition than the Control condition, after correcting for multiple comparisons using the Hommel procedure. When examining the model AUCs of individual participants (Fig 2, Bottom Right), this difference appears to mainly be driven by a larger proportion of participants who discounted heavily, rather than by a systematic shift downward of all participants. Only 23% (8/35) of Control condition participants had mean estimated AUCs less than 0.5, as compared to 31% (8/26) of Framing condition participants and 44% (17/39) of Gamified condition participants.

**Fig 3:**
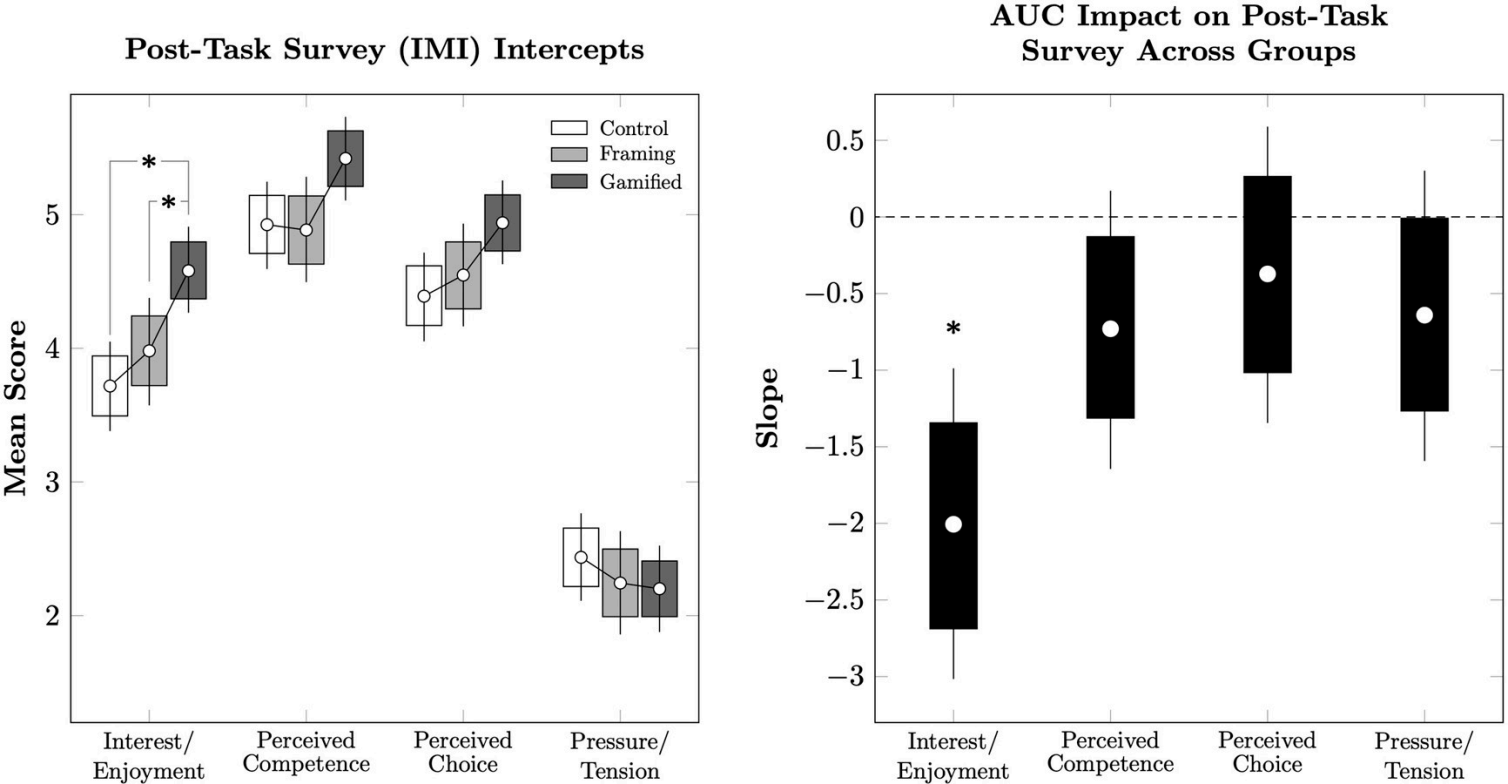
Impact of delay discounting task participation on post-task IMI scores in Experiment 1. Error bars represent the 95% credible interval for the mean, while boxes represent the 80% credible interval. (*) represents a posterior distribution comparison that excludes zero from the 95% credible interval. **(Left).** Mean scores for each of the IMI’s four components in each of the conditions, after controlling for a participant’s model AUC. **(Right).** Mean slopes describing how each of the IMI’s four components change as a function of a participant’s model AUC.

In order to understand participant intrinsic motivation during and after the task, we used a general linear model to predict the four components of the IMI as a function of task condition and each participant’s model AUC. The intercepts of this model are plotted in Fig 3 (Left). After correcting for multiple comparisons using the Hommel procedure, condition did not result in different estimated intercepts in Perceived Competence, Perceived Choice, or Pressure/Tension. However, the Gamified condition had a significantly higher level of Interest/Enjoyment than either the Control condition or the Framing condition. Fig 3 (Right) depicts the estimated impact of participant AUC on each of these four components. Of these, only Interest/Enjoyment varied significantly as a function of AUC. This suggests that participants who derived greater Interest/Enjoyment from the task also displayed lower model AUCs. Thus, greater enjoyment was associated with stronger discounting.

## Experiment 1 discussion

We examined two primary effects: 1) whether gamification affects enjoyment of the core task, and 2) whether gamification impacts DD scores. On average, participants discounted more heavily in the Gamified condition relative to the Control condition, seemingly driven by a greater proportion of individuals discounting more heavily in that condition relative to the others. Interestingly, the Gamified condition engendered the highest interest/enjoyment and varied as a function of participants’ AUC, such that greater intrinsic motivation reported by someone in this domain was associated with greater discounting. There are several ways this might arise. On the one hand, it is possible that participants who are doing something they enjoy display steeper discounting curves. If this is the case, then the use of very bland procedures by the field at large may underestimate discounting rates as compared to those displayed by participants who are engaged in decision-making with more personally rewarding tasks. On the other hand, this effect may reflect a difference in the samples. The GGQ has not been independently validated and may be insensitive to difference between the Control and Gamified groups. Additionally, the groups were relatively small, perhaps masking true differences. Experiment 2 was designed to better address questions as to *who* might be more affected by gamification and in what DD context, thereby remediating these and other design concerns identified after completion of the first study.

## Experiment 2

Experiment 2 was designed to improve experimental control relative to the first experiment. Due to the nature of the Gamified condition in Experiment 1, certain game design elements were implemented that were not feasible in the non-game conditions, leading to potential confounds. The most distinguishing elements were the inclusion of a player avatar (a digital representation of the player) as well as player-controlled movement. In Experiment 2, we decided to remove the avatar entirely as well as the ability to move volitionally. We looked towards the design of visual novels for gamification, a medium that combines interactive fiction (typically represented by branching narratives) with visuals such as character sprites and backgrounds [34]. It is not uncommon for the main character to not be seen in visual novels, giving the illusion that the game is being presented in a first-person format. Several studies have previously examined the use of visual novels in an experimental setting. For instance, Faizal’s [35] study demonstrated that students who used a visual novel to study English scored significantly higher on academic achievement tests than those in the control group. Thus, it was hoped that combining the narrative present in the first study with the framework of a visual novel would provide a more enjoyable experience than the basic control task without potential confounds associated with self-referent avatars and movement.

While participants were not able to predict how long the subsequent delays would be in Experiment 1, they may have discerned a pattern in the amount of money presented, as it always followed the same sequence. If participants became aware of the pattern, they could even predict what the final offer of each time delay was going to be, meaning they could always choose to wait for that final offer if they wished. We modified the randomization of the DD time delays and corresponding payouts and included additional time delays (three and five days) to test delay distributions, a protocol more consistent with other studies in the DD literature [36, 37]. We also improved our assessment of gaming history and interests by using a validated measure of gaming experience, the Game Preference Questionnaire (GPQ) [38], allowing us to better model how individual experience with gaming might relate to susceptibility to gamification and discounting on the task.

### Materials & methods

#### Participants

As in the previous study, all participants were recruited through MTurk from 6/11/18–9/16/18. One hundred eighty-nine participants, ranging from ages 22 to 72, completed the study. The majority of the participants fell between the ages of 31 to 40 (*n* = 80) and over half identified as male (*n* = 108). The sample also included of 79 women and well as one participant who identified as Other in addition to one participant who chose not to answer. While the sample was predominantly White (*n* = 116), it also included Asian or Pacific Islanders (*n* = 57), Black/African Americans (*n* = 9), Latinx/Hispanics (*n* = 2), participants who identified as Other (*n* = 2), and participants who preferred not to answer (*n* = 3).

Using the same consent framework as in Experiment 1, participants provided written consent based on a broad summary of the study, and were compensated $1 for their participation. All procedures, stimuli, and survey materials were approved by the Reed College IRB to assure ethical treatment of participants (protocol no. 2017-F1).

#### Measures

All self-report measures described for Experiment 1 were replicated in Experiment 2, with the exception of the GGQ that was replaced by the GPQ. This measure is a 10-item self-report scale that has been utilized to classify gamers based on their game preferences and habits. The measure then provides a formula that classifies participants as best matching one of four categories: casual gamers (CG), well-rounded gamers (WRG), hardcore gamers (HG), and non-gamers (NonG) [39].

The DD procedure was expanded to five time delay sections: one day, three days, five days, one months, and six months, resulting in four sets of relative DD pairs ($5 - $1000, $400 - $800, $3 - $600, and $2–400). A randomized set of DD choices was chosen from the aforementioned pairs for each time delay, and the pairs were presented on the left and right side of the screen (also randomized), with unelectable arrows placed in-between. To be more analogous to the standard DD administration, both conditions used the word “dollars” rather than using the abstract “gold” used in Experiment 1.

#### Procedures

Participants were randomly assigned to one of two conditions: Control (*n* = 94) or Gamified (*n* = 94). The control condition (Fig 1, Bottom Left) provided instructions and then asked the participant which amount of currency (represented by dollars) they would rather have. In contrast to the first experiment’s Gamified task where participants were given a choice of avatar and allowed to explore, this Gamified condition emulated a visual novel (Fig 1, Bottom Right). Consequently, while the player was able to interact with the world via menu-based commands, there was no avatar to manipulate. The narrative in the gamified version was roughly the same as that presented in the first study, although with a few additions to compensate for the extra time delay. Participants completed the entirety of the study online through SurveyMonkey. Anonymized data from Experiment 2 is available here {https://psyarxiv.com/6xcbe/}.

## Results

Analysis of the GPQ assigned each participant to one of four categories of gamer. Our sample achieved reasonably good coverage of these four types, with double-digit counts of each category in our two task conditions: casual gamers (*n* = 61), well-rounded gamers (*n* = 52), non-gamers (*n* = 42), and hardcore gamers (*n* = 34). Additionally, an analysis of the UPPS-P yielded no significant differences between task conditions; details are included in the electronic supplement.

**Fig 4:**
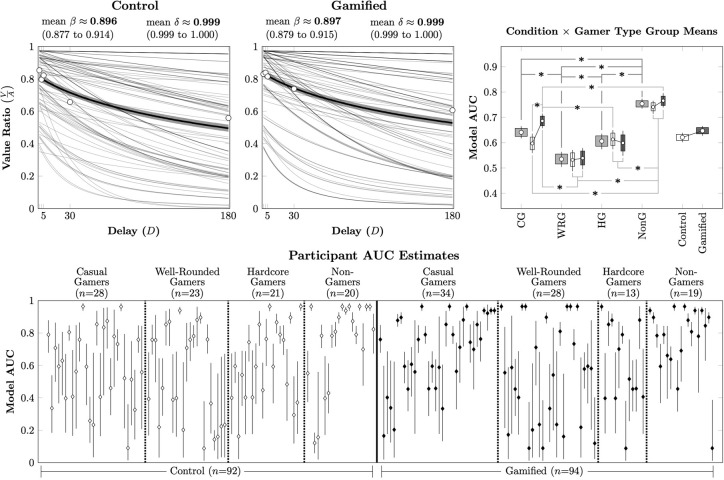
Discounting behavior across participants in Experiment 2. Error bars represent the 95% credible interval for the mean, while boxes represent the 80% credible interval. (*) represents a posterior distribution comparison that excludes zero from the 95% credible interval. **(Top Left & Center).** Posterior means for each participant’s estimated behavior, based on the quasihyperbolic discounting model in each of the two conditions. Thin gray lines represent participant estimates, whereas the thick black line represents the mean across that condition, with shading depicting the 95% credible interval of that mean. Also plotted are the empirical means across participants for the estimated indifference point at each delay. **(Top Right).** Mean parametric AUC across participants for each condition and subdivided according to the gamer type that best describes participants. **(Bottom).** Estimated parametric model AUC for each participant, based on the posterior parameter estimates of the quasihyperbolic distribution.

Fig 4 (Top Left) depicts the mean quasihyperbolic discounting functions for each of the 92 participants in the Control condition, while Fig 4 (Top Center) depicts the discounting functions for the 94 participants in the Gamified condition. Both conditions displayed similarly broad ranges of individual discounting patterns, but the conditions nevertheless yielded nearly identical mean discounting parameters.

Fig 4 (Top Right) shows the bootstrapped mean model AUC for participants, as a function of condition, GPQ category, and their intersection. After controlling for multiple comparisons using the Hommel procedure, there was no overall difference in discounting between the Control and Gamified conditions. However, there were substantial differences in discounting as a function of gamer category. Well-rounded gamers discounted the most, followed by casual and hardcore gamers, whereas non-gamers discounted the least. Only casual gamers showed a difference in discounting as a function of the task, doing so more in the Control condition than in the Gamified condition. That said, every gamer category in every condition displayed a range of discounting behaviors, as depicted by the participant estimated model AUCs in Fig 4 (Bottom).

**Fig 5:**
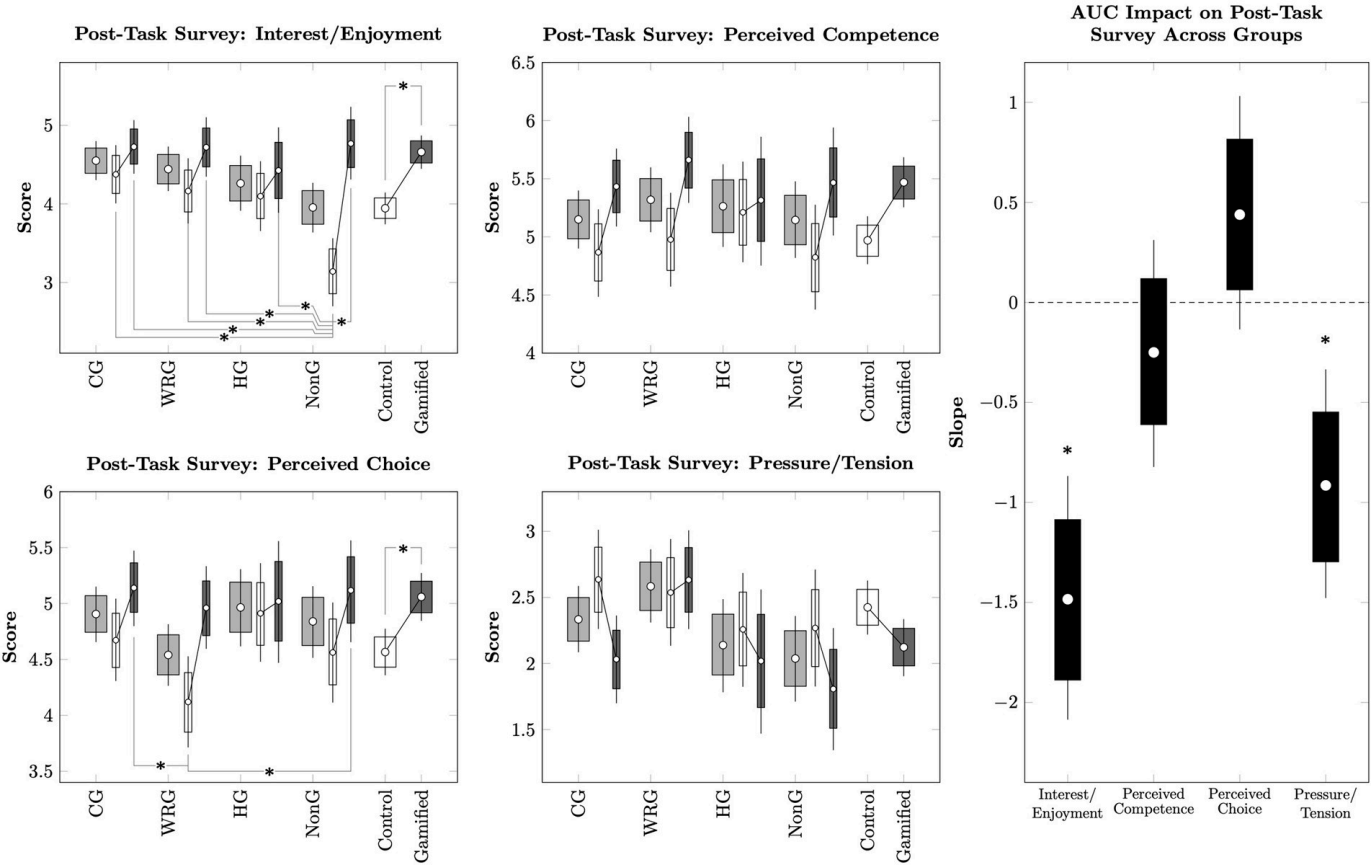
Impact of delay discounting task participation on post-task IMI scores in Experiment 2. Error bars represent the 95% credible interval for the mean, while boxes represent the 80% credible interval. (*) represents a posterior distribution comparison that excludes zero from the 95% credible interval. **(Left).** Mean scores for each of the IMI’s four components in each of the conditions and subdivided according to the gamer type that best describes participants, after controlling for a participant’s model AUC. **(Right).** Mean slopes describing how each of the IMI’s four components change as a function of a participant’s model AUC.

As in Experiment 1, a multivariate regression simultaneously evaluated the four components of the IMI with respect to condition and participant AUC. Additionally, the four gamer categories were included in this analysis. The intercepts of this model are depicted in Fig 5 (Left & Center). Taken overall, participants rated the Gamified condition higher than the Control condition for Interest/Enjoyment and Perceived Choice. With regards to the intersection of condition and gamer category, the most dramatic effect was that non-gamers in the Control condition reported significantly lower Interest/Enjoyment than any other pairing (after correcting for multiple comparisons using the Hommel procedure), whereas non-gamers in the Gamified condition enjoyed the task about as much as all other participants.

Participant model AUC was significantly predictive of Interest/Enjoyment and Pressure/Tension. As such, participants who showed stronger discounting (and thus lower AUC scores) reported higher values than those who did not discount as much.

## Experiment 2 discussion

The aims of Experiment 2 were to replicate the result of increased enjoyment within Experiment 1 without impacting participant’s performance on the DD task and to determine who might be more susceptible to gamification’s impact on intrinsic motivation. While the Gamified condition yielded higher levels of interest/enjoyment (as well as perceived choice), a complex interaction with GPQ’s four categories of game player was also observed that resulted in substantial differences in both discounting and interest/enjoyment. On the one hand, amount of game-playing was strongly predictive of degree of overall discounting, a result consistent with video games appealing to those with high levels of sensation-seeking and impulsivity. Non-Gamers tended to discount the least. However, an unexpected result was that it was non-gamers whose enjoyment most improved as a function of gamification, despite showing comparable levels of discounting in both conditions. This appears to resolve the concern from Experiment 1 that enjoyment and discounting might be directly connected: the nearly-two-point increase in enjoyment by Non-Gamers would have been accompanied by a drop in AUC if that were the case. Setting this complexity aside, however, participants nevertheless appeared to be more engaged with the task in the Gamified condition, without this coming at the expense of their discounting performance differing appreciably from the Control condition. In this larger sample, gamification did not appear to impact the strength of discounting but reliably gave participants greater enjoyment and perceived agency while completing the task. The large improvement to motivation seen in non-gamers demonstrates that these benefits would not be limited to only those who have experience with game aesthetics; if anything, this aspect of our results is consistent with generalized benefit across a wide range of potential participants.

## Conclusions

We investigated whether gamification of a DD task would impact task motivation and performance. Experiment 1 indicated that interest/enjoyment for the task improved when full game elements were included; however, gamification increased discounting in that condition as compared with control, albeit to a limited degree. Using a more modest gamification strategy in Experiment 2, DD performance was uniform across conditions with greater interest/enjoyment and perceived choice reported in the Gamified condition relative to Control. However, these findings masked unexpected and complex differences when gaming history was considered. Participants without gaming experience had the lowest interest and enjoyment in the Control condition but were indistinguishable from other gamer types in the Gamified condition. Participants who discounted the most endorsed greater intrinsic motivation in domains of interest/enjoyment and pressure/tension than those who discounted less.

These finding add to the existing literature on framing, gamification, and the implementation of the DD task. Our findings support Lieberoth’s [20] framification hypothesis as we demonstrated that modest framing changes, such as presenting the choices in the style of interactive fiction, are sufficient to increase enjoyment of the DD task. While more complicated aspects of gamification, like an avatar whose movement whose movement is under player control, also improve motivation, caution should be exercised to ensure that such additions do not substantially change task performance, potentially limiting the validity of the task [22]. Our gamification of the DD task, particularly in Experiment 2, resulted in little to no impact on the strength of discounting, similar to results reviewed by Lumsden et al. [21]. Our findings regarding the interaction of gaming history with motivation and discounting also has implications for the broader literature on media consumption and gaming. Dividing participants into different varieties of game player in the second experiment is consistent with the findings of Weinstein et al. [9], suggesting that individual preferences for certain forms of media may be predictive of their overall impulsivity, with well-rounded gamers showing the strongest propensity for discounting and non-gamers displaying the weakest propensity for doing so. Just as Rachlin [40] notes that actors behave in different ways on stage than they do off the stage due to different sets of discriminative stimuli, those with past experience in gaming may also act differently when interacting with a game. For instance, those with experience in competitive games might naturally be primed to act in a more competitive manner (e.g., seeking a high score) when interacting with a game.

**Fig 6:**
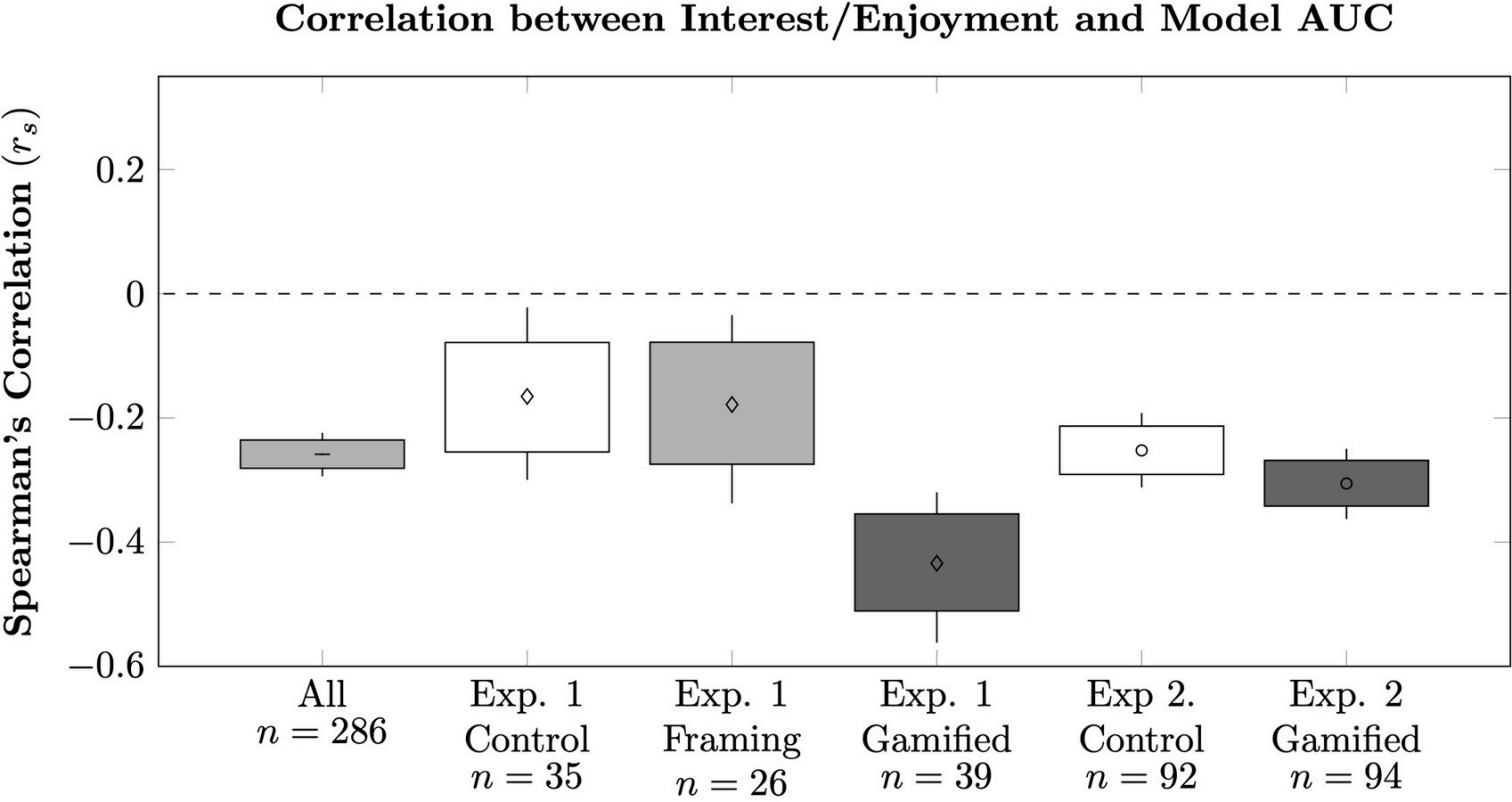
Correlation between model AUC and reported level of interest/enjoyment in both experiments. Boxes represent bootstrapped 80% confidence intervals, whereas whiskers represent 95% intervals. For this analysis, model AUCs for Experiment 1 were recalculated using the longer maximum delay from Experiment 2 in order to make the AUCs comparable across experiments.

However, the individual differences within both experiments consistently pointed to a negative association between the strength of discounting (as measured by AUC) and reported levels of task Interest/Enjoyment (as measured by the IMI). These effects can be seen as global slopes in Fig 3 (right) and Fig 5 (right). While the effect of gamification on the Non-Gamers in Experiment 2 suggests that one cannot simply boost discounting by boosting enjoyment, we felt it was important to verify that this effect was not driven by participants in some conditions. We measured the AUC/IMI correlation for each condition in isolation, and also in pooled analysis that combined both experiments. Estimates of these AUC/IMI correlations are reported in Fig 6. In all cases, a consistently negative correlation was observed. These suggest that despite our between-group comparisons of AUC yielding only small differences in discounting, the variation *within* group varied consistently with level of interest; participants who found the task dull tended to discount very little, whereas those who reported that the task was highly interesting/enjoyable tended to discount substantially more. This effect could contribute to the faster discounting reported by individuals with alcohol and other drug use disorders when asked to consider their substance of choice as the reward: such patients, by diagnostic definition, should find obtaining such outcomes very motivating.

Additional limitations of this work must be noted. While our use of an online data collection format allowed for recruitment of a more diverse age range than typically found in laboratory studies in the area, it also gives rise to variability in the administration of the tasks that cannot be assessed. Participants recruited online may differ in terms of their familiarity with tasks presented and characteristics germane to this investigation (e.g., impulsivity, familiarity of games) than those typically used in DD studies. That said, Schluter, Kim, and Hodgins [39] performed an evaluation of MTurk participants that was roughly contemporaneous with our data collection periods, and found that performance on the DD task was both logically consistent and showed within task reliability with the similarly administered Balloon Analogue Risk Task. While the ages represented in our sample were broader than typical undergraduate samples, our participants were predominantly white, a continued problem with psychological research.

In their traditional implementation, DD tasks can be long, repetitive, and uninteresting [7, 41], factors that may negatively impact data quality [42, 43]. Gamification might be effective in combatting motivational issues for tasks like DD. It is important to recognize that the duration of a DD task is a balancing act between two incompatible aims: (1) to collect as much data as possible in order to maximize confidence in a participant’s estimate, and (2) to keep the task as short as possible to reduce the risk of participants quitting or responding thoughtlessly to “get through” the task. Even though the present study did not find large overall differences in discounting between conditions, we feel that the higher reported level of interest/enjoyment is still important information for those designing new tasks. After all, a more engaging task can be extended to include additional delays and reward amounts, and such a task can also be run more easily with populations who are especially impatient and impulsive. While our DD protocol was relatively brief compared to other studies of DD (in order to allow time for our other questionnaires to be completed), an important future direction would be to make this comparison in a longer DD task, during which a participant’s flagging motivation could pose a greater risk to data quality. Maintaining participant motivation for long enough to get a good estimate of their discounting has clear implications for those deploying DD in a clinical setting rather than a research setting, since clinical evaluations are focused on estimates for single individuals and cannot rely on group averages to reduce residual error. We expect assessment of impulsivity in children would especially benefit from gamification, although this remains a subject for future research.

Deterding et al.’s [15] definition of gamification involves isolating singular mechanics of games and incorporating them into non-game contexts, a definition criticized for not fully encapsulating why people enjoy games [44]. Players do not return to games simply because of a collection of individual elements, like scores or leaderboards; games are complex systems with which players interact. Our findings support the complexity of the task-user interaction in the DD context and the importance of framing in how scientists construct cognitive tasks. How tasks are framed, whether that be through gamification or not, has the potential improve the external validity of our laboratory procedures and participant motivation to perform study tasks with sustained attention and in good faith. That said, caution must be exerted to maintain the key elements of the tasks and data analysis to allow comparison across studies.

It also remains to be seen whether gamification differs in its impact on other kinds of discounting, either parametrically or with respect to theoretical interpretation. For example, a gamified DD task that presents the players with a fictional narrative premise may reveal different kinds of social judgments than one that asks participants to consider real-world scenarios. While it is typical for DD tasks to invite participants to consider what they would prefer in light of hypothetical offers, it is not clear the Control and Gamified conditions represent the same kind of imagination on the part of participants. This difference may impact judgments. “What would you prefer?” may be a different question from “What do you imagine your character would prefer?” By the same token, while DD effects using real-world commodities have been demonstrated [8], it is not clear whether participant will make comparable judgments about fantasy gold, magic crystals, or cybercredits, even if their level of interest is high. The use of “gold” as a currency in Experiment 1 but not in Experiment 2 may play a part in why discounting was stronger only in the Gamified condition of Experiment 1.

Further work will need to be done to validate that gamified DD results have comparable real-world predictive qualities to those observed in traditional DD measures. Additionally, recent work suggests that modifying the DD paradigm to include gamified elements may impact participant performance in dyadic vs individual contexts in unexpected ways [14]. As noted above, playing a game creates a social context in which one may behave in a more competitive, cutthroat manner because “it’s just a game” rather than truly mimicking out of game behavior.

Per the work of Rachlin [45, 46], a broad array of human behavior can be understood when viewed through the lens of discounting. The challenge is determining the complex set of environmental parameters that impact discounting functions, modeling those in the laboratory while considering the ecological validity of such endeavors.

## Supporting information

S1 Data (R)

S2 Data (TXT)
